# The incidence and clinical analysis of non-melanoma skin cancer

**DOI:** 10.1038/s41598-021-83502-8

**Published:** 2021-02-22

**Authors:** Magdalena Ciążyńska, Grażyna Kamińska-Winciorek, Dariusz Lange, Bogumił Lewandowski, Adam Reich, Martyna Sławińska, Marta Pabianek, Katarzyna Szczepaniak, Adam Hankiewicz, Małgorzata Ułańska, Jan Morawiec, Maria Błasińska-Morawiec, Zbigniew Morawiec, Janusz Piekarski, Dariusz Nejc, Robert Brodowski, Anna Zaryczańska, Michał Sobjanek, Roman J. Nowicki, Witold Owczarek, Monika Słowińska, Katarzyna Wróbel, Andrzej Bieniek, Anna Woźniacka, Małgorzata Skibińska, Joanna Narbutt, Wojciech Niemczyk, Karol Ciążyński, Aleksandra Lesiak

**Affiliations:** 1 Department of Proliferative Diseases, Nicolaus Copernicus Multidisciplinary Centre for Oncology and Traumatology, ul. Pabianicka 62, 93-513 Łódź, Poland; 2 Department of Bone Marrow Transplantation and Hematology-Oncology, Pathology, Maria Sklodowska-Curie National Research Institute of Oncology, Gliwice, Poland; 3 University of Technology, Faculty of Medicine, Rolna 43, 40-555 Katowice, Poland; 4 Clinical Department of Maxillo-Facial Surgery, Frederic Chopin Provincial Specialist Hospital in Rzeszów, Rzeszow, Poland; 5 Department of Dermatology, University of Rzeszów, Rzeszow, Poland; 6 Department of Dermatology, Venereology and Allergology, Medical University of Gdańsk, Gdańsk, Poland; 7 Department of Surgical Oncology, Nicolaus Copernicus Multidisciplinary Centre for Oncology and Traumatology, Łódź, Poland; 8 Department of Surgical Oncology Chair of Oncology, Medical University in Łódź, Nicolaus Copernicus Multidisciplinary Centre for Oncology and Traumatology, Łódź, Poland; 9 Dermatology Clinic, Military Institute of Medicine in Warsaw, Warsaw, Poland; 10 Centrum Medyczne Bieniek, Wrocław, Poland; 11 Department of Dermatology and Venereology, Medical University of Łódź, Łódź, Poland; 12 Department of Dermatology, Paediatric Dermatology and Oncology Clinic, Medical University of Łódź, Łódź, Poland; 13 Department of Analysis and Strategy, National Health Fund, Warsaw, Poland; 14 Institute of Applied Computer Science, Lodz University of Technology, Łódź, Poland

**Keywords:** Cancer epidemiology, Cancer prevention, Head and neck cancer, Cancer, Oncology, Cancer epidemiology

## Abstract

Non-melanoma skin cancers (NMSCs) are the most common malignancies diagnosed in Caucasian populations. Basal cell carcinoma (BCC) is the most frequent skin cancer, followed by squamous cell carcinoma (SCC). Unfortunately, most European cancer registries do not record individual types of NMSC. To evaluate the incidence of primary BCCs and SCCs regarding age, sex, tumour site and tumour subtype to determine trends in epidemiology of both cancers. Retrospective analysis of BCCs and SCCs diagnosed and treated across seven sites in Poland from 1999 to 2019. We recorded 13,913 NMSCs occurring in 10,083 patients. BCC represented 85.2% of all cases. SCC patients were older than BCC patients (77.1 ± 11.3 years vs. 70.1 ± 12.3 years, *p* < 0.01). The nodular subtype was the most common subtype of BCC, followed by the superficial and infiltrative subtypes. The superficial BCC subtype was more common on photoprotected areas (*p* < 0.01), whereas the nodular BCC subtype occurred on the face (*p* < 0.01). The high-risk SCC subtypes were more common on face compared to low-risk SCC subtypes (*p* < 0.01). BCC and SCC are common malignancies developing at various ages and anatomical sites. These data underline the need for better registration policies regarding NMSC in order to improve prevention and treatment strategies for these tumours.

## Introduction

Non-melanoma skin cancers (NMSCs) are the most common human malignancies, with steadily rising incidence. The term NMSC refers to all non-melanoma malignant neoplasms affecting the skin. The main types of NMSC, basal cell carcinoma (BCC) and squamous cell carcinoma (SCC), account for about 99% of all NMSCs^1^. Other NMSCs include Merkel cell carcinoma, sebaceous carcinoma, apocrine adenocarcinoma and other rare tumours^2,3^. BCC is the predominant type, with a BCC to SCC ratio between 1:1 and 10:1 depending on the population, ethnic group and sex^4–6^. Although NMSC are 18–20 times more frequent than cutaneous melanoma, there is little epidemiological data for those tumour types^7–9^. Only a few studies have examined the separate incidence of BCCs and SCCs, because in most European countries different types of NMSC are not distinguished from each other in national cancer registry data. Any tumour diagnosis based on histopathology and site is coded according to the International Classification of Diseases 11th Revision (ICD-11). Cutaneous melanoma is coded as C43, therefore the data for this diagnosis is reliable. A non-homogeneous NMSC group receives a single code (C44) to cover all NMSCs, therefore separate data on BCCs, SCCs, and other malignancies of the skin is not available making separate diagnoses of NMSCs difficult to count and assess accurately.

We retrospectively collected, processed, and analysed data on the incidence of separate diagnoses within NMSC group across seven sites in Poland. The aim of the study was to analyse the incidence of primary BCCs and SCCs depending on the site of the lesion, sex and age of the patients to determine epidemiological trends and characteristics of both tumour types.

## Patients and methods

We retrospectively searched histopathological databases for records between 1999 and 2019 in multiple tertiary oncology and dermatology centres in Poland, including Gdansk (north Poland), Gliwice (south Poland), Lodz (two sites, central Poland), Rzeszow (southeast Poland), Warsaw (central Poland), and Wroclaw (southwest Poland). All consecutive patients aged 18 years old and above (those centres do not treat children) with histopathologically confirmed BCC or SCC were included in the study. The database and methods were conducted similarly to our previous study^10^.

The National Comprehensive Cancer Network (NCCN) guidelines were followed to classify the tumour subtype. High-risk BCC subtypes included: infiltrative, micronodular, morpheaform, mixed with aggressive subtype (tumour consisting of more than one subtype, of which at least one is an aggressive subtype) and BCC with carcinosarcomatous differentiation. Low-risk BCC subtypes included: nodular, superficial, fibroepithelial, keratotic and mixed (nodular with superficial). High-risk SCC subtypes included: desmoplastic, acantholytic, carcinosarcomatous and adenosquamous. Low-risk SCC subtypes included: keratoacanthomatous and verrucous.

BCC with mixed histologic subtypes is defined as tumour that is composed of two or more growth patterns within the same lesion. For mixed cases the following rules for classification have been followed: when aggressive subtype predominated in the lesion, the tumour was defined with the most high-risk subtype that was mentioned; when non-aggressive subtype predominated aggressive subtype, the tumour was defined as mixed with aggressive subtype; when tumour consisted of two non-aggressive histologic subtypes it was defined as mixed (nodular with superficial). If there was a discordance between the initial biopsy specimen subtype and the wide excision, then the tumour subtype was classified based on wide excision.

The population studied was fairly homogenous in regards to ethnic group. Data included patients diagnosed in national health centres as well as in private hospitals. Patients’ age and sex, site of the cancer, and histopathological diagnosis were recorded. Recurrent tumours were identified and excluded by comparison with data from earlier diagnoses. Patients were divided into 5-year age intervals for further analysis. The data were analysed statistically by analysis of variance with post-hoc comparisons, the Student’s t-test, and χ^2^ test with Yates correction, if required. Statistical significance was observed for *p* values of less than 0.01.

We also extracted data regarding incidence rates of NMSC between 1999 and 2019 from the Polish National Health Fund database registries using C44.0–C44.9 of ICD-10 coding and compared them to our data^10^.

The Human Research Ethics Committee of the Medical University of Lodz, Poland approved the study (RNN/209/18/KE). All institutions the subjects’ information was sourced from obtained the informed consent. The data used encrypted identification of the subjects, thus written consent was not required from the patients. All procedures and methods were conducted in accordance Declaration of Helsinki^10^.

### Compliance with ethics guidelines

Approval for this study was obtained from the Human Research Ethics Committee of the Medical University of Lodz, Poland (RNN/2019/18/KE). All methods and procedures were conducted in accordance with the relevant guidelines and regulations as well as with the updated Declaration of Helsinki.

## Results

**Figure 1:**
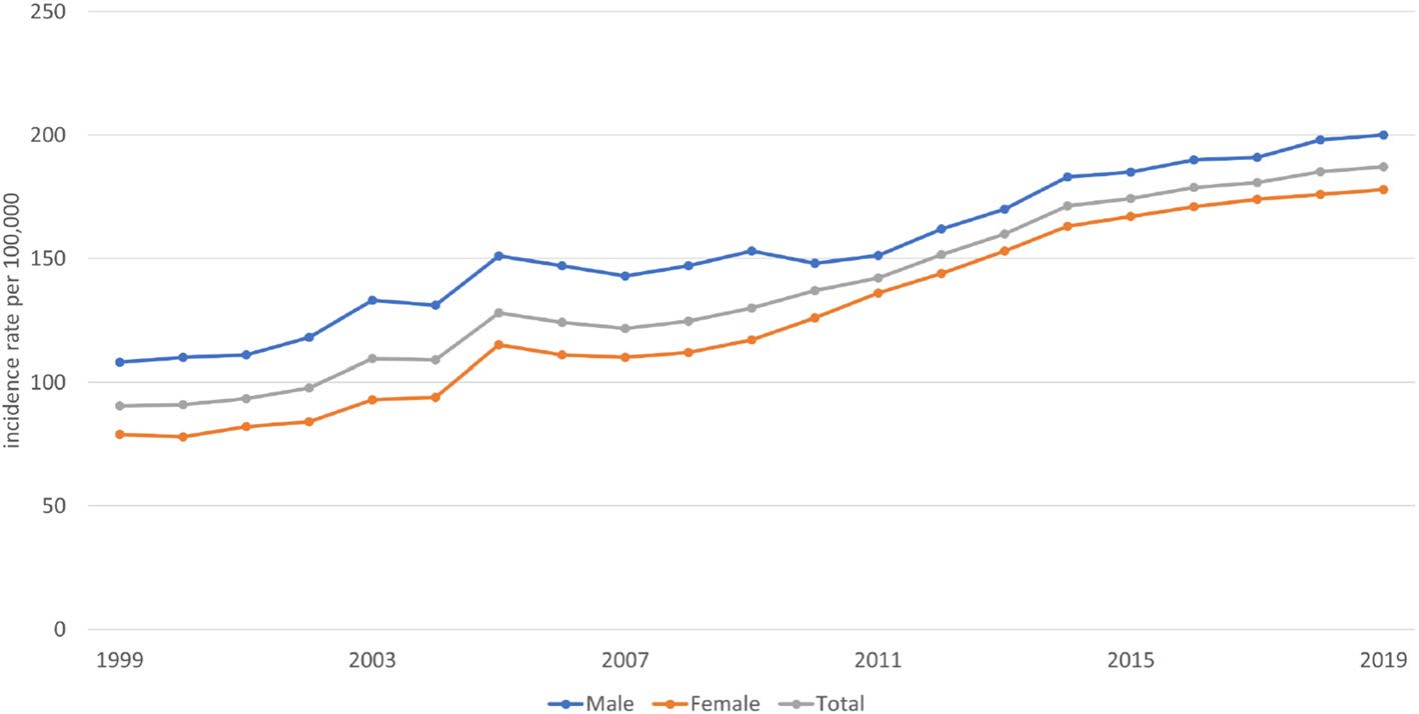
Incidence of cases diagnosed using ICD-10 code C44 between 1999 and 2019 according to the Polish National Health Fund per 100,000 person-years. ICD-10: International Classification of Diseases, 11th Revision.

The data gathered from the Polish National Health Fund database showed an increasing trend of NMSC diagnoses between 1999 and 2019. The annual growth (5–7%) of incidence rates of C44 cases was observed between 2005 and 2014. The trend continued after 2014, although the growth rate slowed down with the annual increase rate not exceeding 2%. Figure 1 presents the incidence rates of cases classified as C44 per 100,000 person-years between 1999 and 2019 in relation to gender. The data gathered in our multicentre study confirmed that the incidence rates of BCCs and SCCs are growing.

**Figure 2:**
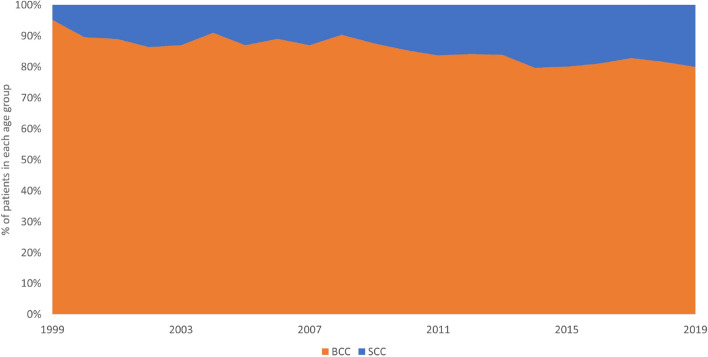
Annual BCC to SCC ratio between 1999 and 2019.

Over the 21-year study period, 10,083 patients were diagnosed with at least one excised and histologically confirmed BCC or SCC. Twenty-three percent of patients had more than one NMSC, resulting in a total of 13,913 NMSC cases (6899 men and 7014 women, ratio 0.98:1), including 11,848 BCCs and 2065 SCCs (BCC:SCC ratio 5.7:1; *p* < 0.01). Figure 2 presents annual BCC to SCC ratio. SCC comprised only 10% of all NMSC in 2000, but this figure was increasing gradually each year where SCC comprised 20% of all NMSC in 2019.

**Figure 3:**
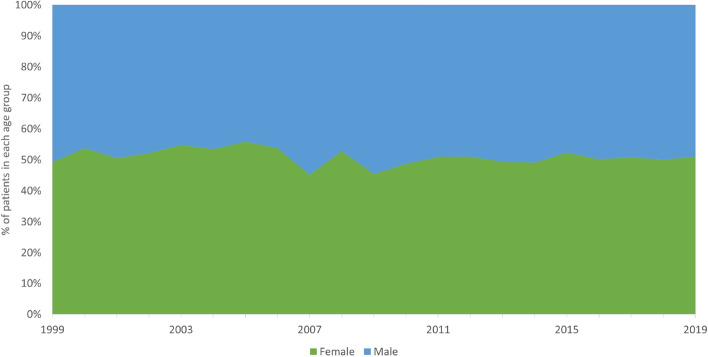
Male and female incidence rate for NMSC relation between 1999 and 2019.

The mean age of patients with BCC was 70.1 ± 12.3 years (range 18–101 years), whereas of patients with SCC was 77.1 ± 11.3 years (range 19–102 years) (*p* < 0.01). Figure 3 presents male to female ratio for NMSCs which did not show any significant annual differences between genders over the analysed period.

There were 5881 men and 5967 women with BCC (ratio 0.99:1). The mean age of men was 70.9 ± 11.4 years and that of women was 71.4 ± 11.9 years (*p* = 0.28).

**Figure 4:**
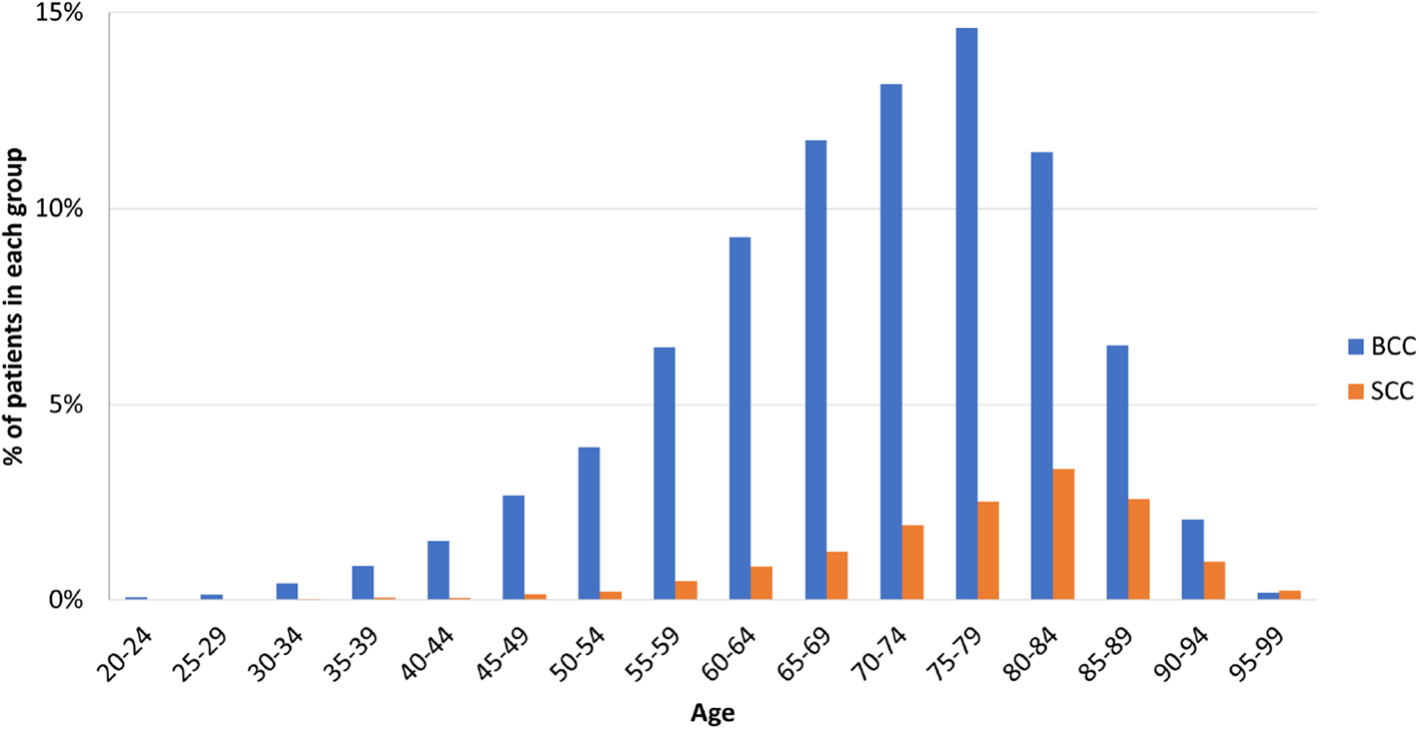
Age-specific incidence rates for NMSC (n = 13,913). SCC patients were significantly older than BCC patients (*p* < 0.01). *NMSC* non-melanoma skin cancer, *SCC* squamous cell carcinoma; *BCC* basal cell carcinoma.

There were 1,018 men and 1,047 women with SCC (ratio, 0.97:1). The mean age of men was 75.9 ± 12.2 years and that of women was 77.2 ± 12.1 years (*p* < 0.01). Figure 4 presents the age-specific distributions for BCCs and SCCs.

**Figure 5:**
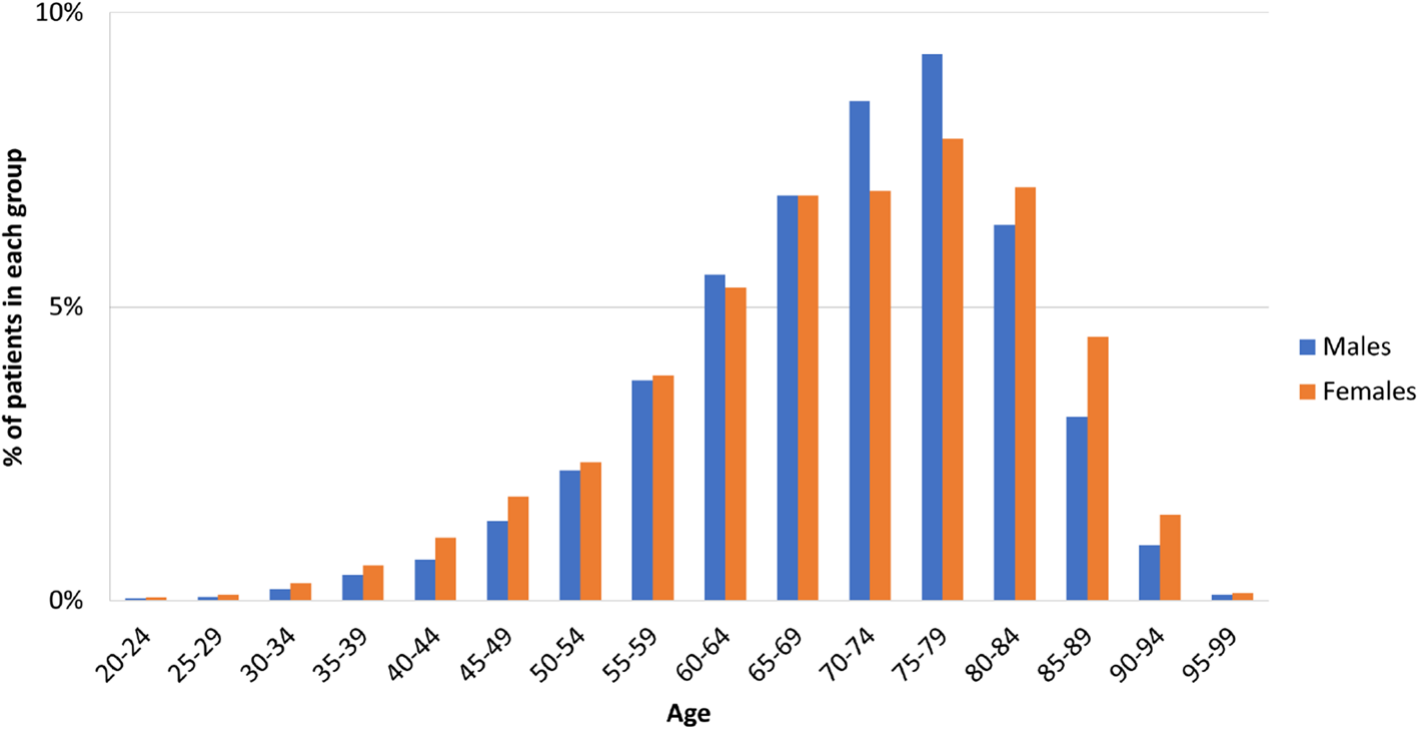
Age-specific incidence rates for NMSC in relation to sex; n = 13,913. In patients 18–44 years old, there was a significant predominance of women (*p* < 0.01). In patients 45–69 years old, there was no predominance in sex (*p* = 0.93). In patients 70–79 years old, there was a significant predominance of men (*p* < 0.01). In patients 80–102 years old, there was a significant predominance of women (*p* < 0.01).

There was a predominance of women (*p* < 0.01) in the youngest group of NMSC patients (18–44 years). In patients 45–64 years, no significant difference in sex was observed (*p* = 0.96). In patients 65–79 years, a significant predominance of men was observed (*p* < 0.01), and in the oldest patients (80–102 years), women were significantly more prevalent (*p* < 0.01). Figure 5 presents the age-specific distributions of NMSCs in relation to gender.

**Figure 6:**
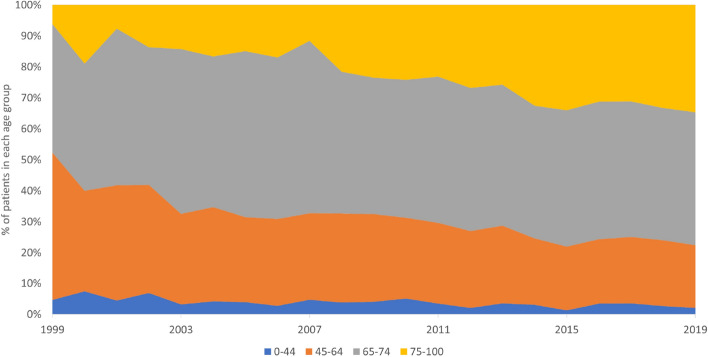
Age-specific incidence rates for NMSC between 1999 and 2019.

During the analysed period, the highest increase in incidence rates for NMSC was observed in the oldest group (over 75 years), while patients in age group between 65 and 74 years showed stable incidence rates. Figure 6 presents annual incidence rates in different age groups.

A single histologically confirmed NMSC was observed in 7786 patients. In 2300 patients, multiple carcinomas were diagnosed. There were two, three, four, and more than four carcinomas in 1564, 389, 165, and 182 patients, respectively. There was no statistically significant relationship between sex and multiple lesions (*p* = 0.47).

**Figure 7:**
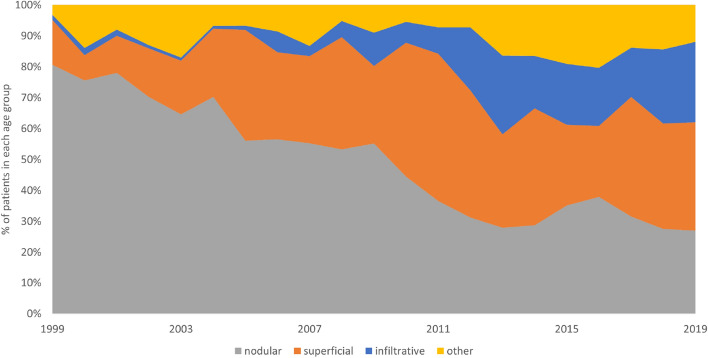
Distribution of BCC subtypes between 1999 and 2019.

SCC subtype was registered in 976 cases including low-risk SCC in 814 cases (412 women, 402 men) and high-risk SCC in 162 cases (90 women, 72 men). Information regarding the subtype of BCC was registered in 5389 cases. Nodular (including the nodular and ulcerative subtype) BCC was the most frequent BCC subtype for both men and women (n = 2369, 44.0%; 1182 cases in men and 1187 cases in women). The other subtypes of BCC were superficial (n = 1641, 30.5%), infiltrative (n = 758, 14.1%), mixed superficial with nodular (n = 182, 3.4%) and mixed with aggressive subtype (n = 61, 1.1%), basosquamous (n = 181, 3.4%), adenoid (n = 25, 0.5%), pigmented (n = 23, 0.4%), micronodular (n = 14, 0.3%) and other (n = 135, 2.5%). Superficial BCCs were more common in women than in men (*p* < 0.01), whereas other BCC subtypes demonstrated no statistically significant relationship between sex and histopathological subtype (nodular BCC, *p* = 0.79; infiltrative BCC, *p* = 0.38; other BCC subtypes, *p* = 0.19) Table 1 presents the SCC and BCC subtypes according to two-tiered classification of BCCs (high and low risk subtypes) in relation to the patients’ gender and tumour’s site^11,12^. During the analysed period a significant increase in infiltrative subtype was observed (threefold increase between 2010 and 2019). Incidence of superficial BCC subtype increased by 1% annually, while nodular subtype incidence declined over the above period. Figure 7 presents distribution of BCC subtypes between 1999 and 2019.

**Table 1:** BCC and SCC subtypes sites depending on the sex of the patients.

	Low-risk SCC	High-risk SCC	Low-risk BCC	High-risk BCC
**Men**				
Face	225 (61%)^a^	47 (85%)^a^	549 (57%)^a^	350 (78%)^a^
Trunk	46 (13%)	0 (0%)	263 (27%)	37 (8%)
Upper limb	32 (9%)	2 (4%)	62 (6%)	13 (3%)
Lower limb	41 (11%)	0 (0%)	44 (5%)	4 (1%)
Scalp	11 (3%)	2 (4%)	20 (2%)	27 (6%)
Neck	13 (4%)	4 (7%)	51 (5%)	16 (4%)
n/d	34	17	1059	85
all	402	72	2048	532
**Women**				
Face	275 (67%)	82 (92%)	549 (57%)	364 (81%)
Trunk	27 (7%)	0 (0%)	248 (26%)	43 (10%)
Upper limb	23 (6%)	1 (1%)	48 (5%)	7 (2%)
Lower limb	57 (14%)	2 (2%)	70 (7%)	7 (2%)
Scalp	2 (0%)	1 (1%)	25 (3%)	18 (4%)
Neck	12 (3%)	3 (3%)	55 (6%)	12 (3%)
n/d	4	1	1161	87
all	412	90	2166	548

Data are presented as n (%).

*n/d* not defined, *BCC* basal cell carcinoma, *SCC* squamous cell carcinoma.

^a^High-risk BCCs and SCCs were most common on the face (*p* < 0.01).

The mean age of the patients differed depending on various BCC subtypes. Patients with superficial BCC were slightly younger (67.7 ± 11.5 years), than patients with other subtypes (69.3 ± 12.7 years), and the difference was statistically significant (*p* < 0.01). Moreover, women with superficial BCC were younger than men with the same BCC subtype (67.0 ± 13.7 years vs. 68.6 ± 12.2 years; *p* < 0.01). On the other hand, patients with basosquamous BCC (72.2 ± 11.3 years), nodular BCC (69.4 ± 12.7 years), infiltrative BCC (71.1 ± 12.3 years) were on average older than patients with other subtypes.

Anatomical distribution of NMSCs was collected in 10,587 cases (8962 BCC cases and 1625 SCC cases). In both sexes, BCC and SCC lesions were located mainly on the face. Overall, BCC occurrence on the face was statistically more frequent when compared to SCC (*p* < 0.01). The second most frequent sites were the trunk for BCC (14.8%) and the lower limbs for SCC (11.8%). Table 2 presents the clinical characteristics of patients with BCC and SCC.

**Table 2:** Clinical characteristics of patients with BCC and SCC.

	BCC	SCC
Total number of lesions	11,848	2065
Mean age, years, total	70.1^a^	77.1^a^
Mean age, years, male	70.1	75.9^b^
Mean age, years, female	70.1	78.2^b^
Sex ratio (F/M)	1.01	1.03
**Location**		
Face	6500 (73%)	1036 (64%)
Trunk	1372 (15%)	160 (10%)
Upper limbs	316 (4%)	128 (8%)
Lower limbs	303 (3%)^c^	192 (12%)^c^
Scalp	186 (2%)	44 (3%)
Neck	285 (3%)	65 (4%)

*BCC* basal cell carcinoma, *SCC* squamous cell carcinoma, *F/M* female to male ratio.

^a^The mean age of patients differed significantly between SCC and BCC patients (*p* < 0.01).

^b^The mean age of SCC patients differed significantly between men and women (*p* < 0.01).

^c^SCC was significantly more common on the lower limbs than BCC (*p* < 0.01).

BCCs on the face were located most often on the nose (29.7%). Other areas on the face included the eye (16.2%), cheek (15.8%), temple (12.0%), forehead (11.4%), earlobe (9.1%), lips (3.2%), chin (1.4%), nasolabial folds (0.62%), and the jaw-line (0.62%).

While nodular and infiltrative BCC was located mainly on the face, superficial BCCs were most frequent on the trunk (*p* < 0.01). Lesions on the trunk were significantly more common in younger patients (67.3 ± 13.5 years), whereas lesions on the face occurred mainly in older patients (72.1 ± 11.9 years; *p* < 0.01). Women developed lesions on the trunk and upper limbs at a younger age than men (*p* < 0.01), whereas lesions on the face were more common in younger men than women (*p* < 0.01).

## Discussion

NMSCs are the most common skin malignancies with rising incidence all over the world, mainly because of increased exposure to UV radiation and ozone depletion with sun-seeking behaviours and higher cumulative UV dose, connected with increased longevity, as contributing factors^13,14^. As already mentioned, the exact incidence of BCCs and SCCs is difficult to establish. Majority of cancer registries record these cancers under the term NMSC, instead of recording separate diagnoses. Previously published data indicated that BCCs comprise about 80% of all NMSCs and this incidence continues to rise^13,14^, which is consistent with the results of our multicentre study.

Although skin cancers may develop in children and young adults^15^, it is essentially a disease of the older population^16,17^. The European population is an aging one and as a consequence, a notable increase in the occurrence of all cancers is observed^18,19^. About 80% of cancers are diagnosed in patients older than 55 years, although the increase is observed in all age groups, regardless of sex. We observed an increasing trend for NMSC incidence in our study, which is consistent with data gathered from the Polish National Health Fund database.

Most patients with BCCs (95%) are between 40 and 79 years of age^20,21^. Our study also confirmed that SCC develops more frequently in older patients. Since 2012, the increase in the incidence of both BCC and SCC was observed among the older population. Interestingly, our findings showed that while the SCC incidence raised significantly also in younger population, the growth was significantly higher in the elderly, which is in line with the results of previous studies^22,23^.

The rising trend in the incidence of BCCs and SCCs may be due to numerous factors. An improved consciousness of cutaneous malignancies in the overall population due to skin cancer prevention campaigns may have led to more skin diagnoses and examinations of previously undiagnosed NMSC. Moreover, all physicians are more aware of skin cancers, which may also lead to an increased likelihood of skin cancers, including NMSC, being diagnosed^24,25^. Moreover, since 1990 air travel has become widely available and affordable to the general population in Poland, allowing people to travel to sunny destinations, where the skin is exposed to higher than usual and acute doses of UV radiation. Another factor in raised incidence of NMSC has been a substantial increase in sunbed use, particularly in younger population, over the past two decades. The sunbed use reached its peak of popularity between the 1990s and 2000s, but regulatory changes including a minimum age restriction of 18 years have been introduced only recently^26^.

Despite the overall increase in the incidence of both BCC and SCC between 1999 and 2019, it is noteworthy that BCC rates for people 40 years old and younger showed no substantial increase in our study. Studies conducted almost 20 years ago showed raised incidence of NMSC in women younger than 40^27,28^. Currently, these women are much older than 40, and we could have expected an increase in the incidence of NMSCs in this group of patients. Moreover, women who were 40–50 years of age in 2018 were those who, in the 1990s and 2000s, had used sunbeds extensively. Considering that the main factor of the lifetime potential for developing skin cancer is the sun exposure in childhood and adolescence^29^, although adult sun exposure also plays a role^30–33^, it could be hypothesised that in the following decades the frequency of BCCs in patients younger than 40 may stabilise or even decrease, providing proper introduction of skin cancer prevention programs reducing childhood and teenage sun exposure.

The incidence of BCCs rose more sharply in women than in men and in patients younger than 50 and older than 80. BCCs in men were most often reported in patients between 50 and 80 years old. Data reported by Eurostat in 2017 showed that women in the European Union live on average 5 years and 3 months longer than men^34^. Most studies indicate a higher risk for NMSC development in the elderly, which could explain the greater proportion of cancer in women in the older age group.

Older studies found that more men than women develop skin cancer^35,36^. This is probably due to the fact, that man used to receive more exposure to sunlight during outdoors work and sport activities. However, there have been recent changes in attitudes in recreational sun exposure and many people, regardless of gender, receive a considerable dose of UVR from a very early age. This has already led to a reversal of the sex ratio between patients with BCC as fewer young men than young women develop skin cancer. BCC located on the trunk is also more common in young people, especially women^37^.

In our study, most tumours were located on the face, followed by the trunk, lower and upper extremities, neck and scalp. The site distribution in described population is consistent with those previously reported^38^. In accordance with previous studies, BCCs were predominantly located in the chronically UV-exposed regions^39^.

In our study, men were not more susceptible than women to develop either BCC or SCC apart from the ear site as 16% of the facial BCCs occurring on the ear were found in men, whereas only 5% in women. It is more common for women to have hair covering the ears, and therefore this anatomic site is more protected from UV. More than 60% of all diagnosed subtypes of BCC on the ear were of aggressive subtype, which is in concordance with data gathered by Jarell et al.^40^. This information could help physicians to ensure that the suspected lesions on the ear are promptly biopsied and treated.

We revealed that nodular subtype is the most common subtype of BCC, but in our study, it accounted for only 44% of all BCC cases, not 60–80% indicated by other studies^38,39^. Recent data indicates that BCC is not homogenous and that various histological types present different clinical behaviour and could have a different background. It was also shown that certain histological subtypes of BCC develop more often in specific anatomical sites. In our study superficial BCC occurred predominantly on the trunk in both sexes, especially in younger patients. These observation supports the hypothesis of intermittent sun-exposure for development of superficial BCC, as the exposures to acute, higher UV doses are more likely to develop during youth, especially for women, and decrease as people age^40,41^.

In contrast, nodular BCC most frequently occurs on the face^37,39^. Our results support previous hypothesis that chronic cumulative sun exposure is more related to the nodular type of BCC (similarly to SCC)^42–45^. We also revealed that SCC, particularly on the face, was more frequently diagnosed in older patients. The median age of SCC patients was higher than that of BCC patients. Interestingly, SCC was presented more commonly on the lower limbs compare to BCC.

The incidence of high-risk BCC, including the infiltrative, micronodular, and morpheaform subtypes, was higher in our study than in previously reported European and Australian data^42–45^. Moreover, the most frequent location for infiltrative lesions was on the face, where surgical removal is more difficult, and the risk for incomplete excision is higher^45,46^.

A significant increase in infiltrative subtype was observed in all sites (threefold increase between 2010 and 2019). The underlying cause is not clear. However, recently improved biopsy techniques could improve the diagnosis of high-risk subtypes. Aggressive subtypes are infiltrative and often grow in the deep portion of the tumour. Hence, small biopsy specimens centred topographically may miss deeper infiltrative component. Moreover, the environmental changes related to the ultraviolet light exposure, ozone depletion in various parts of the world and genetic predisposition could be also etiological factors for increase in the incidence of infiltrative BCC^21^. However, this topic requires further research.

Our study showed that BCC with mixed histologic subtypes was observed in 4.5%. Other studies have indicated incidence of mixed subtypes between 11 and 39%^47–50^. The significantly lower number of mixed subtypes could be caused by the methodology where mixed cases have been classified as most high-risk subtype that was mentioned. BCCs showing mixed subtypes with a predominating aggressive pattern were registered not as a mixed subtype but as an aggressive subtype.

In most cases only the first histopathologically confirmed NMSC per patient is registered^17^, but as our results show, multiple lesions are relatively common. In our study multiple lesions were observed in 23% of patients, and 7% of patients had three or more lesions. Other studies revealed that around 30% of patients will develop subsequent skin cancers within 5 years of the first NMSC diagnosis^17^. In a 2002 survey 25% of people with diagnosed NMSC had at least two skin cancers treated in a 12-month period^51^. In our study, multiple primary NMSCs were common, and almost 9% of patients developed a second NMSC within 2 years. This is consistent with the results of previous studies^51,52^.

## Conclusions

We present rare epidemiological data of different types of NMSC diagnosed and treated in central Europe in form of a large series of surgically treated BCCs and SCCs over a period of 12 years. The most frequent site of the lesions was face; however, we observed an increasing trend in the incidence of truncal tumours in younger patients. The distribution of histopathological subtypes confirmed the predominance of nodular BCCs, followed by superficial and infiltrative BCCs.

Looking at the worldwide epidemiological trends of NMSC, it is obvious that there is an urgent need to control the increasing incidence of NMSC and subsequent socioeconomic burden. Studying and understanding current epidemiological trends of NMSC is crucial to achieve an early and adequate control of those common skin cancers. We need to respond more actively to this “NMSC epidemic” to stabilise and ultimately decrease BCC and SCC rates in the future. Effective primary preventative skin cancer strategies need to be developed to avoid future impact on the Health Services.

## Data Availability

The data that support the findings of this study is available from the corresponding author upon request.

## Abbreviations

BCC: Basal cell carcinoma
BSC: Basosquamous cell carcinoma
NMSC: Non-melanoma skin cancer
SCC: Squamous cell carcinoma
