# Author Correction: The incidence and clinical analysis of non‑melanoma skin cancer

**DOI:** 10.1038/s41598-021-94435-7

**Published:** 2021-07-28

**Authors:** Magdalena Ciążyńska, Grażyna Kamińska-Winciorek, Dariusz Lange, Bogumił Lewandowski, Adam Reich, Martyna Sławińska, Marta Pabianek, Katarzyna Szczepaniak, Adam Hankiewicz, Małgorzata Ułańska, Jan Morawiec, Maria Błasińska-Morawiec, Zbigniew Morawiec, Janusz Piekarski, Dariusz Nejc, Robert Brodowski, Anna Zaryczańska, Michał Sobjanek, Roman J. Nowicki, Witold Owczarek, Monika Słowińska, Katarzyna Wróbel, Andrzej Bieniek, Anna Woźniacka, Małgorzata Skibińska, Joanna Narbutt, Wojciech Niemczyk, Karol Ciążyński, Aleksandra Lesiak

**Affiliations:** 1 Department of Proliferative Diseases, Nicolaus Copernicus Multidisciplinary Centre for Oncology and Traumatology, ul. Pabianicka 62, 93-513 Łódź, Poland; 2 Department of Bone Marrow Transplantation and Hematology-Oncology, Maria Sklodowska-Curie National Research Institute of Oncology, Gliwice, Poland; 3 University of Technology, Faculty of Medicine, Rolna 43, 40-555 Katowice, Poland; 4 Clinical Department of Maxillo-Facial Surgery, Frederic Chopin Provincial Specialist Hospital in Rzeszów, Rzeszow, Poland; 5 Department of Dermatology, University of Rzeszów, Rzeszow, Poland; 6 Department of Dermatology, Venereology and Allergology, Medical University of Gdańsk, Gdańsk, Poland; 7 Department of Surgical Oncology, Nicolaus Copernicus Multidisciplinary Centre for Oncology and Traumatology, Łódź, Poland; 8 Department of Surgical Oncology Chair of Oncology, Medical University in Łódź, Nicolaus Copernicus Multidisciplinary Centre for Oncology and Traumatology, Łódź, Poland; 9 Dermatology Clinic, Military Institute of Medicine in Warsaw, Warsaw, Poland; 10 Centrum Medyczne Bieniek, Wrocław, Poland; 11 Department of Dermatology and Venereology, Medical University of Łódź, Łódź, Poland; 12 Department of Dermatology, Paediatric Dermatology and Oncology Clinic, Medical University of Łódź, Łódź, Poland; 13 Department of Analysis and Strategy, National Health Fund, Warsaw, Poland; 14 Institute of Applied Computer Science, Lodz University of Technology, Łódź, Poland

Correction to: *Scientific Reports* 10.1038/s41598-021-83502-8, published online 22 February 2021

The original version of this Article contained errors in the Affiliations.

Affiliation 2 was incorrectly given as ‘Department of Bone Marrow Transplantation and Hematology-Oncology, The Maria Skłodowska-Curie Memorial Cancer Centre and Institute of Oncology, Branch in Gliwice, Gliwice, Poland’. The correct affiliation is listed below.

Department of Bone Marrow Transplantation and Hematology-Oncology, Maria Sklodowska-Curie National Research Institute of Oncology, Gliwice, Poland.

Additionally, Affiliation 3 was incorrectly given as ‘Department of Tumor Pathology, The Maria Skłodowska-Curie Memorial Cancer Centre and Institute of Oncology, Branch in Gliwice, Gliwice, Poland’. The correct affiliation is listed below.

University of Technology, Faculty of Medicine, Rolna 43, 40-555, Katowice, Poland.

The original Article has been corrected.

